# Plasma metabolomics of *Mycoplasma synoviae* infection in SPF White Leghorn hens by liquid chromatography-tandem mass spectrometry

**DOI:** 10.1186/s13567-025-01494-z

**Published:** 2025-03-22

**Authors:** Chun Wang, Qing Wang, Yang Li, Zhaoyang Wang, Bo Hou, Naiji Zhou, Weitao Cui, Sishun Hu, Yuncai Xiao, Wanpo Zhang, Hongbo Zhou, Zili Li, Zutao Zhou

**Affiliations:** 1 College of Veterinary Medicine, Huazhong Agricultural University, Wuhan, China; 2 State Key Laboratory of Agricultural Microbiology, Huazhong Agricultural University, Wuhan, China; 3 Key Laboratory of Preventive Veterinary Medicine in Hubei Province, Huazhong Agricultural University, Wuhan, China; 4 Institute of Animal Husbandry and Veterinary Medicine, Fujian Academy of Agricultural Sciences, Fuzhou, China

**Keywords:** *Mycoplasma synoviae*, laying hens, infection model, plasma metabolomics, LC–MS/MS

## Abstract

*Mycoplasma synoviae* (*M. synoviae*) is a major bacterial pathogen that causes serious economic losses in the global poultry industry. Systemic changes in specific pathogen free White Leghorn egg-laying hens after *M. synoviae* infection were investigated using intra-tracheally inoculated animals. Samples were collected 10 days post-infection (dpi) (204-day-old) and 52 dpi (246-day-old). Infection caused air sac lesion, footpad swelling and oviduct atrophy. The qPCR and in situ hybridization showed that bacteria colonized the trachea and oviduct, and that bacterial loads in the magnum and uterus were significantly higher than in the infundibulum and isthmus. Histopathological examination revealed increased tracheal mucosal thickening accompanied by inflammatory cell infiltration, and that tubular glands of the uterus were edematous or dissolved. Infection also induced decreased egg production and eggshell strength, and eggshell apex abnormalities appeared at 14 dpi. Plasma metabolomics of hens analyzed by liquid chromatography-tandem mass spectrometry showed 168 and 128 differentially-expressed metabolites (DEM) at 10 and 52 dpi, respectively. Pathway analysis revealed that DEM at 10 dpi were enriched in five distinctive pathways: regulation of the actin cytoskeleton, neuroactive ligand-receptor interaction, sphingolipid metabolism, gap junctions, and necroptosis. In contrast, DEM at 52 dpi were enriched in fifteen pathways involved in steroid hormone biosynthesis, ferroptosis, the calcium signaling pathway, apelin signaling pathway, progesterone-mediated oocyte maturation, and oocyte meiosis. Combined metabolic analysis demonstrated that changes in ethylsalicylate, nicotinamide, (3-Methoxy-4-hydroxyphenyl) ethylene glycol sulfate, sphingosine-1-phosphate (d18:1), carnitine C24:6, and 15(R)-prostaglandin E1 correlated the best with *M. synoviae* infection. This study provides new insights into understanding pathogen mechanisms and signposts novel treatments for *M. synoviae* infection in poultry.

**Supplementary Information:**

The online version contains supplementary material available at 10.1186/s13567-025-01494-z.

## Introduction

*Mycoplasma synoviae* (*M. synoviae*) is one of the most important microbial pathogens that affects poultry from both clinical and economic perspectives. *M. synoviae* is transmitted horizontally and vertically by eggs, and infection induces respiratory disease, airsacculitis, and synovitis in chickens and turkeys [1, 2, 3]. Chronic and subclinical conditions are characteristic of *Mycoplasma* infection [4]. In view of the high prevalence of *M. synoviae* infection in layers [5], the impact of *M. synoviae* on laying hens has prompted studies aimed at deciphering the modes of transmission, infection, and therapeutic strategies for this pathogen.

Since the first report of eggshell apex abnormality (EAA) caused by *M. synoviae* in the Netherlands in 2009 [6], numerous instances of the condition have occurred globally [7–13]. EAA is linked to decreased egg production and quality, and is responsible for substantial economic losses [6, 14]. Dutch [6], Italian [11], Australian [9], Brazil [10] and Polish [12] strains of *M. synoviae* have been implicated in EAA, but information is scarce regarding the effects of Chinese isolates of *M. synoviae* on eggshell quality.

Metabolomics are used effectively in diagnosing pathologies, identifying therapeutic targets of disease, and for investigating the mechanisms that underpin fundamental biological processes [15]. Plasma, serum, and urine are used widely as sample sources to discover disease markers in clinical metabolomics [16]. Metabolomics are also employed extensively in the study of both infectious and noninfectious diseases in humans and other animals. For example, the principal classes of glycerophospholipids, sphingolipids, and fatty acyls increased in patients with *Mycoplasma pneumoniae* infection, which mainly induced alterations in glycerophospholipid and sphingolipid metabolism [17]. Moreover, significant changes in the host amino acid and fatty acid profiles occur during early stages of *Mycoplasma hyopneumoniae* infection [18]. However, to our knowledge, metabolomics tools have not been employed to study the host responses to *M. synoviae* infection.

In this study, we conducted an experimental infection with specific pathogen free (SPF) White Leghorn egg-laying hens with the aim of identifying the metabolic changes associated with *M. synoviae* pathogenesis. This approach replicated a model of *M. synoviae* infection that leads to abnormal egg production. Liquid chromatography-tandem mass spectrometry (LC–MS/MS) was used to pinpoint the metabolic differences between infected and uninfected laying hens. The findings provide key insights into the pathogenesis of *M. synoviae* infection and suggest new strategies for clinical treatment of this important condition that impacts poultry worldwide.

## Materials and methods

### *M. synoviae* challenge strain

*M. synoviae* MS-XS (Chicken/Hubei/4/2021) was isolated from a diseased chicken in Hubei, China and was cultured in modified Frey liquid medium at 37 ℃ in a 5% CO_2_ incubator (Thermo Fisher Scientific, Massachusetts, USA) [19].

### Experimental design and sample collection

**Figure 1:**
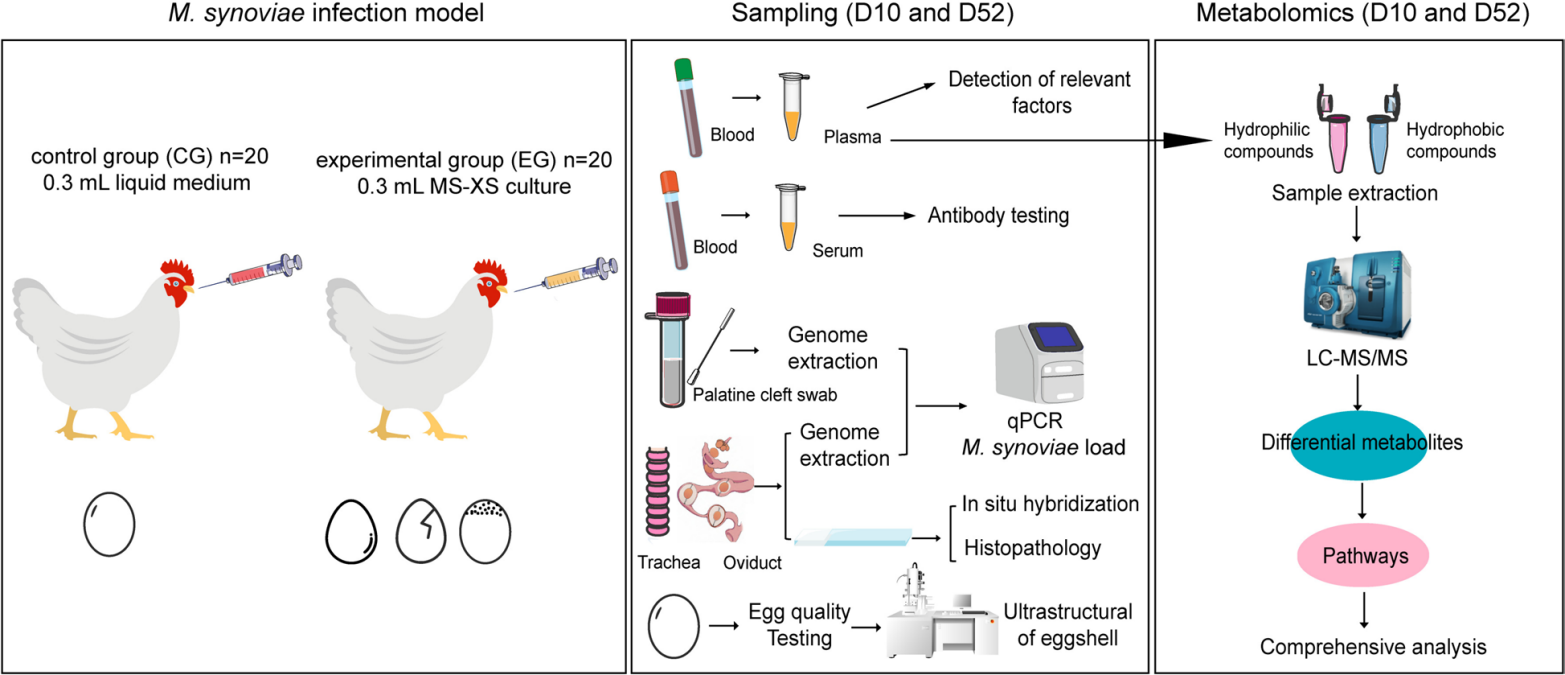
**General workflow of the study**. Hens were inoculated intra-tracheally with *M. synoviae* (left). Sample collection methods (center) and sample processing procedures (right) are illustrated.

Forty 162-day-old SPF White Leghorn egg-laying hens (Jinan Spafas Poultry Co., Ltd., Jinan, China) were housed in isolators under negative pressure (Suzhou Fengshi Laboratory Equipment Co., Ltd., Suzhou, China) with water and a commercial chicken feed (Beijing Keao Xieli Feed Co., Ltd., Beijing, China) ad libitum. The hens were divided randomly into two groups with an average of four isolators (a, b, c, d). The hens in the non-infection group (*n* = 20) in isolators a and b were used as a control group (CG), and the infection group of hens (*n* = 20) in isolators c and d were used as the experimental group (EG). CG hens were inoculated intra-tracheally with 0.3 mL of liquid medium when egg production reached a stable peak period (194 days), whereas EG hens were inoculated with 0.3 mL of MS-XS culture (10^8^ CCU/mL). Hens subsequently were monitored for egg production. At 10 (204-day-old) and 52 dpi (246-day-old), one isolator (*n* = 10) was selected at random from each group, the animals were euthanized, and were examined for air-sac lesions and given a lesion score according to the criteria of Kleven et al. [20]. Trachea, oviduct, and liver samples were collected immediately, frozen with liquid nitrogen, and stored at -80 ℃. Serum samples and swab of palatine cleft were collected and processed for further analysis. The number of copy equivalents of *M. synoviae* per gram of mucus was calculated based on the mucus uptake per flocked cotton swab and assuming that the release of mucus from the swab during washing was 100%. The mucus uptake per swab was determined by weighing individual swabs before and after sampling using a calibrated analytical balance. Plasma samples were collected for metabolomics analysis (Figure 1).

### The qPCR assay for detection of *M. synoviae* loads

Genomic DNA were extracted from trachea and oviduct tissue samples (50 mg) with the TIANamp Genomic DNA Kit (Tiangen, Beijing, China) and from swab samples with the SteadyPure Universal Genomic DNA Extraction Kit (Accurate Biotechnology (Hunan) Co., Ltd., Changsha, China), stored at -20 ℃. Loads of *M. synoviae* were quantified by SYBR Green-based qPCR [21] (Bio-Rad, Hercules, CA, USA). Primers targeted to the vlhA gene (accession no. AF035624.1) (forward: 5’-TTAGCAGCTAGTGCAGTGGCC-3’; reverse: 5’-ACCTGGATTTCCTGATTGGTTTTGAGG-3’) were used for qPCR analysis [21]. The qPCR program was as follows: 2 μL template DNA was added to reaction mixture (20 μL) containing 10 μL 2 × SYBR Green Premix (Accurate Biotechnology (Hunan) Co., Ltd.) and 0.4 μL of each primer pair. The PCR reaction mixtures were subjected to the following conditions: pre-denaturation at 95 ℃ for 30 s, followed by 40 cycles of 95 ℃ for 5 s, 60 ℃ for 30 s. The *vlhA* gene segment of strain MS-XS was subcloned in the pMD18-T vector and transformed into *E. coli* DH5α. Serial tenfold dilutions of positive plasmid from 10^2^ to 10^8^ copies/μL were used to establish a standard curve. The obtained cycle threshold (Ct) values of tested samples were plotted against DNA copy number on the standard curve. A Ct value more than 32 was considered negative for detection.

### Serology

Biochek *M. synoviae* antibody kits (Biochek, Hoofddorp, the Netherlands) were used to determine the prevalence of antibodies in the experimental layer hens. A titer value ≥ 594 was considered positive.

### Exclusion of the main pathogens causing secondary infections

Genomes were extracted (TIANamp Genomic DNA Kit, Tiangen, Beijing, China) from the trachea and liver (*n* = 5 per group) to detect avian pathogenic *Escherichia coli* (APEC, serotypes: O1, O2, O78). RNA was extracted (SteadyPure Quick RNA Extraction Kit, Accurate Biotechnology (Hunan) Co., Ltd., Changsha, China) from the trachea and uterus (*n* = 5 per group) and then cDNA synthesis was performed using a cDNA reverse transcript kit (Vazyme, Nanjing, China) according to the manufacturer’s instructions to detect infectious bronchitis virus (IBV). The primers for APEC [22] and IBV [23] are shown in Additional file 1. The triplex PCR program for APEC (O1, O2, and O78) and PCR program for IBV were as follows: 1 μL template DNA or cDNA was added to the reaction mixture (25 μL) containing 12.5 μL 2 × PCR Taq Mix (Vazyme, Nanjing, China) and 0.5 μL of each primer pair. The PCR reaction mixtures were subjected to the following conditions: pre-denaturation at 95 ℃ for 5 min, followed by 30 cycles of 95 ℃ for 15 s, 55 ℃ for 15 s (APEC) or 52 ℃ for 30 s (IBV), 72 ℃ for 30 s and a final extension at 72 ℃ for 5 min. The PCR products were observed under ultraviolet light after electrophoresis on a 1.5% agarose gel. Biochek IBV antibody kits (Biochek, Hoofddorp, the Netherlands) were used to detect IBV serum antibodies (*n* = 6 per group). A titer value ≤ 833 was considered negative.

### Measurement of plasma cytokines and reproductive hormone concentrations

The concentrations of cytokines interleukin-6 (IL-6) and tumor necrosis factor-α (TNF-α) and reproductive hormones progesterone and E2 estradiol were determined using chicken-specific ELISA kits (Meimian, Jiangsu, China).

### Histopathology and in situ hybridization examination

Trachea and oviduct fragments from the same position of each hen were placed into 4% paraformaldehyde and fixed for three days before being processed for hematoxylin and eosin (H&E) staining and in situ hybridization (ISH) for detection of tissue lesions and *M. synoviae* distribution, respectively. Procedures used to perform H&E staining followed methods described elsewhere [24]. The primers targeted to the *vlhA* gene (forward: 5’-TTAGCAGCTAGTGCAGTGGCC-3’; reverse: 5’-GTAACCGATCCGCTTAATGC-3’) were used for recycling this gene from MS-XS DNA [19]. The probe was synthesized in vitro using DIG-High prime DNA labeling mix (Roche, Basel, Switzerland).

The tissue sections were deparaffinized in xylene for 20 min, followed by rehydration through a series of graded alcohols (100%, 95%, 90%, and 80%; 2 min of each). The sections were incubated in 0.2 M HCl for 20 min in the dark, then washed twice with ultrapure water for 5 min. After heating at 85–95 ℃ for 20 min, the tissues were digested with Proteinase K (15 ng/μL) at 37 ℃ for 15 min and subsequently washed with 2 × SSC. Following dehydration in increasing alcohol concentrations, the sections were incubated with DIG Easy Hyb Granules (Roche) at 37 ℃ for 30 min. Hybridization was performed using a probe (6 ng/μL) in a ThermoBrite S500-24 Hybridization System (StatSpin Abbott, IL, USA) at 95 ℃ for 7 min and then at 37 ℃ for 13 h. The sections were washed with a series of buffers: wash 1 (2 × SSC for 5–10 min at 37 ℃), wash 2 (2 × SSC with 0.1% SDS for 8 min, twice at 37 ℃), and wash 3 (0.5 × SSC with 0.1% SDS for 8 min at 55 ℃). After blocking with a ten-fold diluted solution (Roche) in maleic acid for 45 min at 37 ℃, the sections were incubated with Anti-Digoxigenin-AP Conjugate (Roche; diluted 1:2000 in blocking solution) for 45 min at 37 ℃. The sections were then subjected to wash 1 (maleic acid with 0.3% Tween 20 for 8 min, four times) and wash 2 (0.1 M Tris–HCl, 0.1 M NaCl, pH 9.5 for 8 min). Finally, the signal was developed using NBT/BCIP substrate (Roche).

### Egg quality testing

Eggs produced from all hens at 3 to 10 dpi and 50 to 52 dpi were used for quality testing, including measurements of egg weight, egg shape index, and eggshell weight, strength, and thickness. Longitudinal and transverse egg diameters and egg shape index were measured with vernier calipers, and eggshell strength was assessed using a Tenovo eggshell strength tester (Beijing Tianxiang Technology Co., Ltd., Beijing, China). Eggshell thickness (blunt end, middle end, and tip) was determined with a micrometer and eggs and eggshells were weighed with an electronic balance.

### Ultrastructure observation for measurement of eggshell quality

Two shell samples from the equatorial region of each egg (0.1–0.2 cm^2^) were prepared for ultrastructure examination. One section was washed in ddH_2_O and left to dry at room temperature for examination of the eggshell cross section. The second sample was soaked in ddH_2_O to remove shell membranes and then was immersed overnight in 6% NaClO, 4.12% NaCl, and 0.15% NaOH to remove the remaining tightly bound membrane fibers for analysis of the shell surface. Samples were glued on an aluminum support, metalized with gold, and visualized with a JSM-6390LV scanning electron microscope (JEOL Ltd., Tokyo, Japan).

### Plasma metabolomics profiling

Analysis of plasma untargeted metabolomics was performed by Ultra Performance Liquid Chromatography (UPLC) and tandem mass spectrometry (MS/MS) (LC–MS/MS, Wuhan Metware Biotechnology Co., Ltd., Wuhan, China). To minimize the influence of physiological factors, such as reproductive cycle, on metabolomics results, six hens with the same egg position in the oviduct were selected as experimental subjects in each group. Hydrophilic compounds were extracted by mixing 50 μL of the sample with 300 μL of extraction solution (acetonitrile:methanol = 1:4, v/v), containing internal standards. The mixture was vortexed for 3 min and centrifuged at 10 000 × *g* for 10 min at 4 ℃. The supernatant (200 μL) was collected and stored at −20 ℃ for 30 min, followed by centrifugation at 10 000 × *g* for 3 min at 4 ℃. Aliquots (180 μL) of the supernatant were retained for analysis.

For hydrophobic compounds, 50 μL of the sample was mixed with 1 mL of extraction solvent (methyl tert-butyl ether:methanol = 3:1, v/v) containing an internal standards mixture. The mixture was swirled for 15 min, followed by the addition of 200 μL of water. After vortexing for 1 min, the sample was centrifuged at 10 000 × *g* for 10 min. The upper organic layer (200 μL) was collected and evaporated using a vacuum concentrator. The dry extract was reconstituted in 200 μL of acetonitrile:isopropanol (1:1, v/v) for analysis.

Metabolite analysis was conducted using an ExionLC AD UPLC system coupled with a QTRAP^®^ MS/MS. Hydrophilic metabolites were separated on an ACQUITY UPLC HSS T3 C18 column (1.8 µm, 2.1 mm × 100 mm). Two microlitres of supernatant were injected and eluted at a flow rate of 0.4 mL/min and 40 ℃. The solvent system consisted of A: water (0.1% formic acid) and B: acetonitrile (0.1% formic acid), with a gradient from 95:5 (0 min) to 10:90 (12 min), reverting to 95:5 by 14 min.

Hydrophobic metabolites were separated on a Thermo Accucore^™^ C30 column (2.6 µm, 2.1 mm × 100 mm i.d.) using a 2 μL injection and a flow rate of 0.35 mL/min at 45 ℃. The solvent system comprised A: acetonitrile/water (60/40, v/v, 0.1% formic acid, 10 mmol/L ammonium formate) and B: acetonitrile/isopropanol (10/90, v/v, 0.1% formic acid, 10 mmol/L ammonium formate). The gradient ranged from 80:20 (0 min) to 5:95 (15.5 min), returning to 80:20 by 20 min.

For both analyses, the ESI source was operated at 500 ℃ with an ion spray voltage of 5500 V (positive) and −4500 V (negative). Gas parameters included GS1 at 55 psi, GS2 at 60 psi, and curtain gas at 25 psi for hydrophilic analysis; and GS1 at 45 psi, GS2 at 55 psi, and curtain gas at 35 psi for hydrophobic analysis. The collision gas was set to high for hydrophilic and medium for hydrophobic analyses. Instrument tuning and mass calibration were performed using 10 and 100 μmol/L polypropylene glycol solutions in QQQ and LIT modes, respectively.

### Bioinformatics analysis

Orthogonal partial least-squares discrimination analysis (OPLS-DA) was used to determine differences between experimental groups. Score plots and permutation plots were generated using R package MetaboAnalystR [25]. For two-group analysis, differentially-expressed metabolites (DEM) were determined by variable import in projection (VIP; VIP > 1, extracted from OPLS-DA result) and *P*-value (*P* < 0.05, Student’s *t* test). The data was log transformed (log_2_) and mean centered before OPLS-DA. Identified metabolites were annotated using the Kyoto Encyclopedia of Genes and Genomes (KEGG) compound database and annotated metabolites were then mapped to the KEGG pathway database. The diagnostic power of the model was evaluated by receiver operating characteristic (ROC) analysis. The area under the curve (AUC) is defined as the area enclosed by the coordinate axis under the ROC curve. In general, an AUC of 0.7 to 0.8 is considered acceptable, 0.8 to 0.9 is considered excellent, and greater than 0.9 is considered outstanding [26].

### Statistical analysis

All data are presented as mean ± standard deviation (SD) and were analyzed using SPSS 23.0 (IBM Corporation, Armonk, NY, USA). The data of Ct value and *M. synoviae* load were analyzed with one-way and two-way analysis of variance (ANOVA). The rate of egg production was analyzed by Chi-Squared Test. The concentrations of plasma factors and reproductive hormones were analyzed with Student *t*-test. *P* < 0.05 was regarded as statistically significant. Graphs were generated by GraphPad Prism 8.0 (GraphPad Software, La Jolla, CA, USA) and Adobe Illustrator (Adobe, San Jose, CA, USA).

## Results

### Establishment of a model for SPF laying hens infected with *M. synoviae*

An infection model of *M. synoviae* was established by intra-tracheal inoculation of SPF White Leghorn egg-laying hens with 0.3 mL of MS-XS culture (10^8^ CCU/mL). Necropsy studies show that 6 of 10 EG hens had air sac lesions at 10 dpi and the air sac lesion score was 3.17 (± 0.98). In contrast, only 1 of 10 EG hens had air sac lesions at 52 dpi and the lesion score was 2 (Table 1 and Additional file 2). Oviduct atrophy occurred on both days 10 and 52 after infection, but swelling of the footpads was found only on day 52 (Additional file 2).

**Table 1:** **Gross pathology in each group at 10 and 52 dpi**

Isolator	Group	Sampling time (dpi)	Proportion of air sac lesion (mean ± SD)	Proportion of oviduct atrophy	Proportion of footpad swelling
a	CG	10	0/10 (0)	0/10	0/10
b	CG	52	0/10 (0)	0/10	0/10
c	EG	10	6/10 (3.17 ± 0.98)	3/10	0/10
d	EG	52	1/10 (2)	3/10	2/10

EG: experimental group in which hens were intra-tracheally inoculated with 0.3 mL of MS-XS culture (10^8^ CCU/mL), CG: control group in which hens were intra-tracheally inoculated with 0.3 mL of liquid medium.

### Exclusion of secondary infections caused by APEC (O1, O2 and O78) and IBV

The results are shown in Additional file 3. The PCR electrophoresis results for APEC and IBV show that no nucleic acids from either pathogen were detected in the tissues (Additional file 3). The titer of antibody testing for IBV was < 833 (301.15 ± 238.43), which revealed no secondary infection with IBV (Additional file 3).

### *M. synoviae* infection perturbs egg laying capacity and promotes diverse histopathological changes

**Figure 2:**
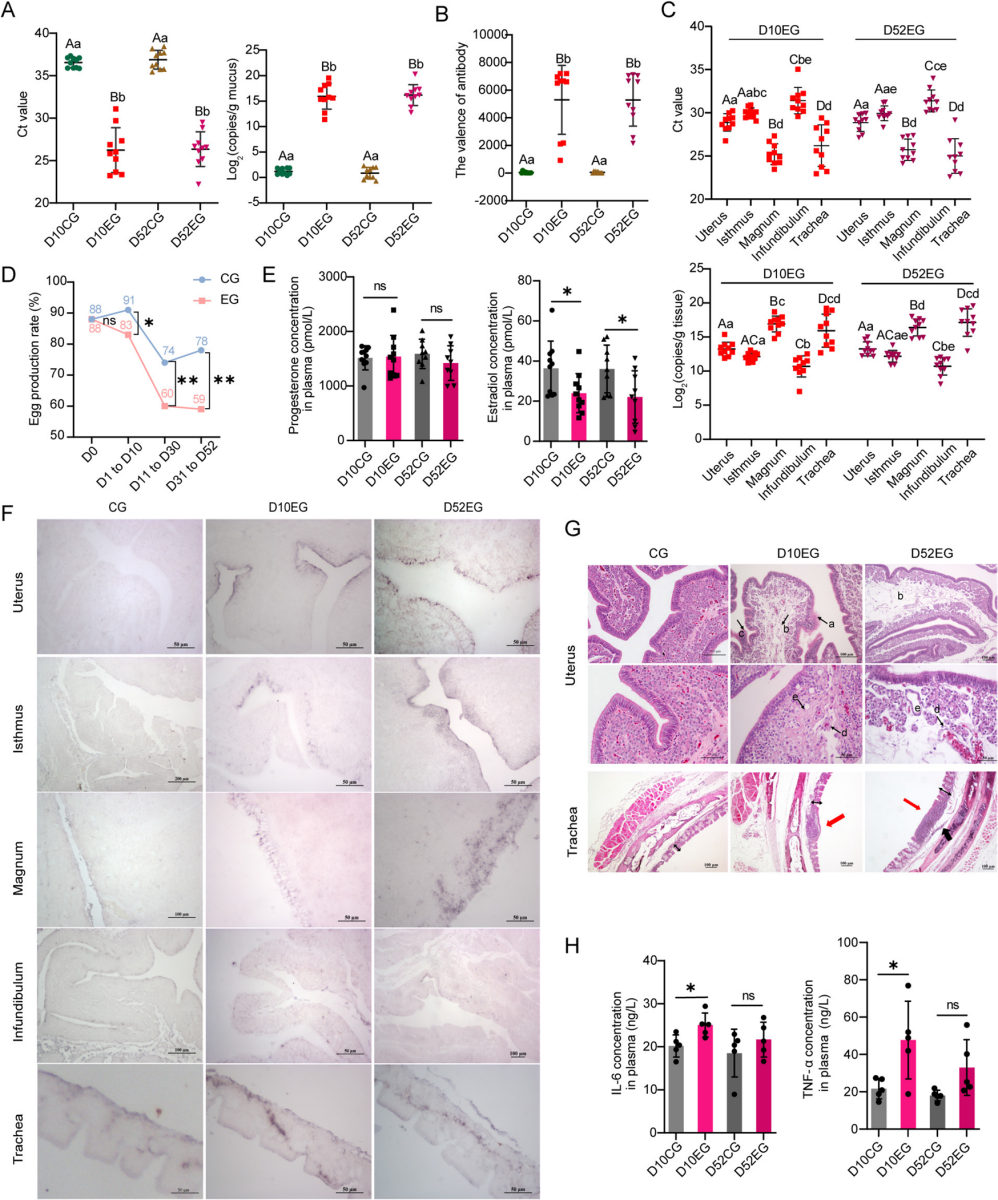
**Effects of** ***M. synoviae* infection on laying hens.** **A** Detection of *M. synoviae* genetic material in palatine cleft swabs. **B** Analysis of serum antibody titer. **C** Detection of *M. synoviae* genetic material in trachea and oviduct of laying hens with *M. synoviae* infection. **D** Statistics of total egg production rate at days 10 and 52 for each experimental group. **E** Analysis of plasma reproductive hormones progesterone and estradiol. **F** The distributions of *M. synoviae* in oviducts and trachea were detected by ISH using *M. synoviae* probes labeled with digoxin. Purple color indicates positive staining of *M. synoviae* nucleic acid. Scale bars indicate 100 μm and 50 μm. **G** Histopathology of uterus and trachea after infection with *M. synoviae* was observed by H&E staining. Scale bars indicate 100 μm and 50 μm. In the uterus, **a** cilia abnormal or fallen off;** b** lamia propria edema;** c** mucosal epithelial layer thickening; **d** degeneration or sloughing off of glandular epithelial; and,** e** tubular gland edema or dissolution. In the trachea, double-headed black arrows indicate mucosal thickness; black thick arrows indicate mucosal layer rupture; and, red arrows indicate inflammatory cell infiltration. **H** ELISA of plasma inflammatory factors IL-6 and TNF-α. The same lowercase letter indicates no significant difference (*P* > 0.05), different lowercase letters indicate significant differences (*P* < 0.05), and different uppercase letters indicate very significant differences (*P* < 0.01). ns, no significant difference (*P* > 0.05); *significant difference (*P* < 0.05); **very significant difference (*P* < 0.01).

The Ct values in EG hens on both 10 and 52 dpi significantly decreased compared with CG hens, and *M. synoviae* loads increased correspondingly (Figure 2A). There was also a significant increase in *M. synoviae* serum antibodies after infection (Figure 2B). Absolute quantitative detection of *M. synoviae* load was observed in four segments of the oviduct (infundibulum, magnum, isthmus, and uterus) and in the trachea of EG hens at 10 and 52 dpi (Figure 2C). It is important to note that *M. synoviae* loads in the trachea and magnum were extremely high compared to other tissues (*P* < 0.01), and that the *M. synoviae* load in the uterus was significantly greater (*P* < 0.05) than in either infundibulum or isthmus (Figure 2C). In addition, a gradual drop in egg production occurred after *M. synoviae* infection. The total egg production rate of EG hens show a significant decrease (8%) in the first ten days of infection compared to CG hens, and then declined further to 60% which was significantly different from CG hens (Figure 2D). Compared with CG hens, there was no change in plasma progesterone in EG animals, but estradiol levels decreased significantly (Figure 2E).

ISH was conducted to investigate further the localization and distribution of *M. synoviae* in diverse segments of the oviduct and trachea. Positive signals for *M. synoviae* show localized distribution in the epithelia of uterus and isthmus at 10 dpi, whereas the signals were distributed uniformly in the epithelia at 52 dpi. *M. synoviae* was distributed evenly in the epithelium of magnum at 10 dpi and, in addition, positive signals indicating agglomerative invasion towards glands or their surroundings were apparent at 52 dpi. *M. synoviae* was not evident in the infundibulum. Positive ISH signals in the trachea were detected on both 10 and 52 dpi (Figure 2F). Histopathological examination was performed on the trachea and the uterus from which the eggshell forms. The mucosal epithelium of the uterus was lined by ciliated pseudostratified epithelium and the lamina propria was filled with tubular glands in CG hens (Figure 2G). However, lesions were detected throughout the infection period in EG hens. Thus, the cilia were abnormal and sloughed off and the mucosal epithelial layer was thickened at both 10 and 52 dpi (Figure 2G). Furthermore, degeneration or sloughing of glandular epithelial cells in the edematous lamia propria and edema or dissolving of tubular glands were also observed at both timepoints in EG hens. The tracheal mucosal thickness of EG hens was greater than that of CG hens and severe infiltration of inflammatory cells into the tracheal lamina propria was apparent (Figure 2G). Finally, compared with the CG animals, the plasma concentration of IL-6 and TNF-α in EG hens increased significantly (*P* < 0.05) at 10 dpi. There also was a general upward trend of these markers at 52 dpi, although this change was not significant statistically (Figure 2H). In summary, infection with *M. synoviae* induces multiple histopathological and immunological changes in laying hens.

### *M. synoviae* infection impairs egg and eggshell quality

**Figure 3:**
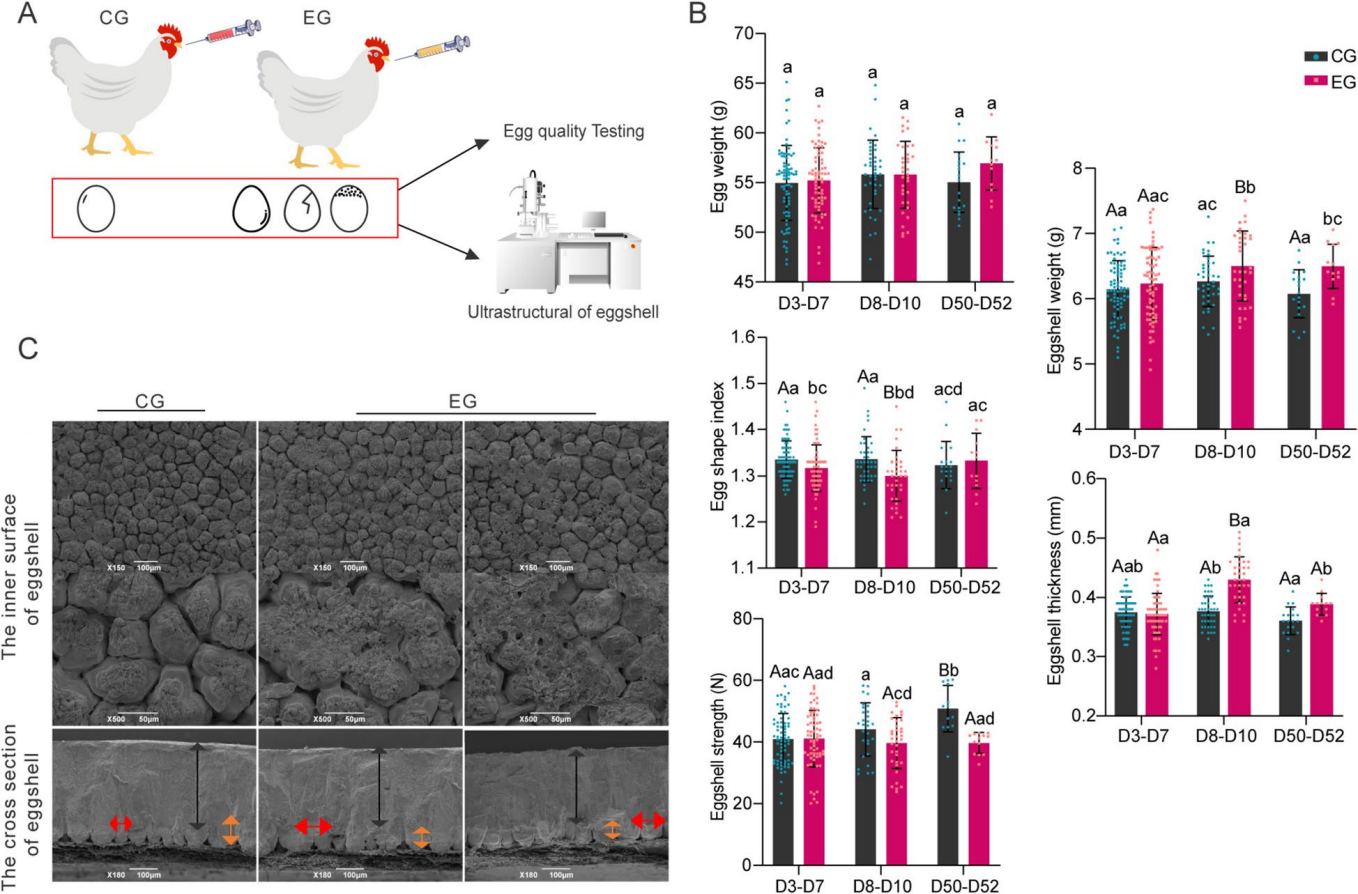
**Effects of* M. synoviae* infection on egg and eggshell quality.** **A** Schematic representation of quality testing for egg and eggshell. **B** Analysis of egg quality at different time periods for each experimental group. The sequence is egg weight, eggshell weight, egg shape index, eggshell thickness, and eggshell strength. The same lowercase letter indicates no significant difference (*P* > 0.05), different lowercase letters indicate significant differences (*P* < 0.05), and different uppercase letters indicate very significant differences (*P* < 0.01). **C** Ultrastructure changes in eggshells after infection with *M. synoviae*. Ultrastructure of the inner surface (bar = 100 μm and 50 μm) and cross section of eggshells (bar = 150 μm). Black arrow, thickness of palisade layer; red arrow, width of mammillary; orange arrow, thickness of mammillary.

EAA is characterized by a distinct zone of weak shell with a roughened surface at the apex of the egg. The first egg with EAA in infected hens was observed at 14 dpi and an EAA of 20–30% was observed thereafter (Additional file 2). The quality of eggs and eggshells was assessed by measurement of egg and eggshell weights, egg shape index, eggshell thickness, eggshell strength, and observation of eggshell ultrastructure (Figure 3A). There was no difference (*P* > 0.05) in weight of EG eggs and CG eggs, but a significant difference (*P* < 0.05) in egg shape index was detectable in the first 10 days of infection (Figure 3B). Furthermore, the eggshell weight and thickness of EG eggs increased significantly (*P* < 0.05) compared to CG eggs seven days after infection, whereas eggshell strength decreased significantly (*P* < 0.05) (Figure 3B). The mammillary body ultrastructure of CG eggs was observed to be normal by electron microscopy and presented a clear honeycomb-like arrangement. However, the mammillary body structure of EG eggs was damaged severely which rendered it difficult to distinguish the boundaries between mammillary bodies. The palisade layer, mammillary layer, and overall thickness of EG eggs were lower than those of control eggs, with a decrease in mammillary thickness and an increase in mammillary width (Figure 3C).

### Changes occur in the plasma metabolome after 10 and 52 days of *M. synoviae* infection

**Figure 4:**
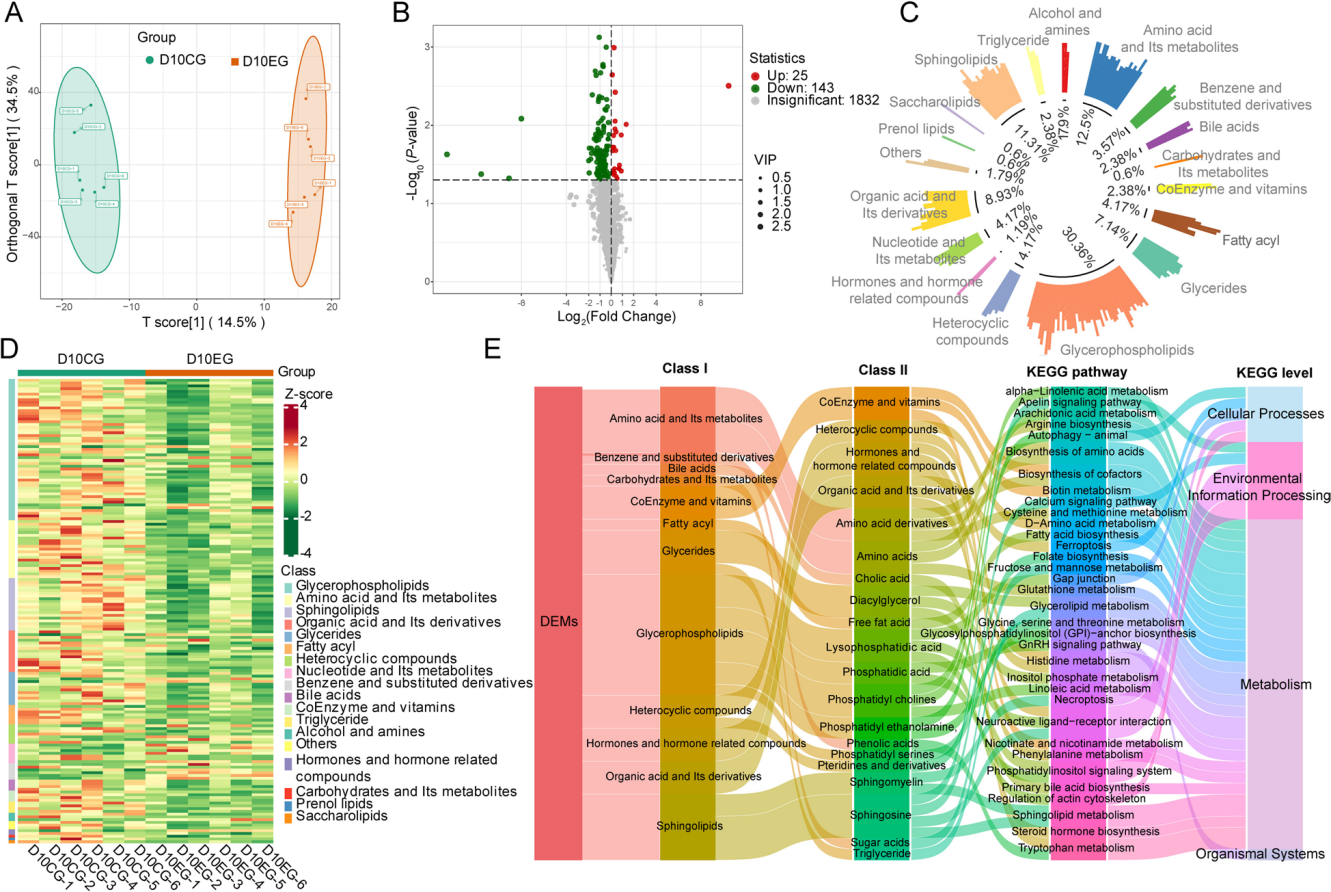
**Analysis of plasma DEM in day 10 EG (n = 6) and CG (n = 6) hens. A** OPLS-DA analysis of DEM shows a clear distinction between CG and EG hens. **B** Volcano plot of metabolites. Significant DEM (VIP > 1 and *P* < 0.05) are in red (upregulated) and green (downregulated), whereas other metabolites are colored in gray. **C** Circle chart of the proportion of primary classification of DEM. **D** Heat map of DEM. Different colors are filled with different values obtained after standardization of relative content (red represents high content, green represents low content). **E** Sankey diagram shows classifications of differential metabolism, as well as KEGG annotations and KEGG classifications.

Metabolomics data were analyzed using the OPLS-DA model and score plots were prepared to discriminate differences in plasma samples from EG and CG hens. The plots from EG and CG animals at 10 dpi were distinct in the OPLS-DA analysis which indicate differences in metabolite profiles of the samples (Figure 4A). DEM were determined by VIP > 1 and *P* < 0.05 (Additional file 4). Twenty-five and 143 DEM were upregulated and downregulated, respectively, in EG plasma compared with CG samples (Figure 4B). Glycerophospholipids, amino acids and metabolites, and sphingolipids accounted for 30.36%, 12.5% and 11.31% of the DEM, respectively (Figure 4C). A heatmap of the original relative content of the DEM identified using screening standards facilitated observation of changes in relative content of metabolites and validated that most DEM in EG plasma were decreased compared to CG plasma (Figure 4D).

The KEGG annotation of the DEM was classified by pathway type to explore the potential function of these metabolites (Additional file 5). The DEM were associated mainly with metabolism, cellular processes, environmental information processing, and organismal systems. KEGG annotation also demonstrated that the main affected metabolic pathways involved lipid metabolism, amino acid metabolism, carbohydrate metabolism, and metabolism of cofactors. At the level of cellular processes, the DEM were implicated principally in autophagy, ferroptosis, necroptosis, gap junction, and regulation of the actin cytoskeleton. The calcium signaling pathway, apelin signaling pathway, neuroactive ligand-receptor interaction, and phosphatidylinositol signaling system were altered at the level of environmental information processing, and the gonadotropin-releasing hormone (GnRH) signaling pathway in organismal systems was affected (Figure 4E).

**Figure 5:**
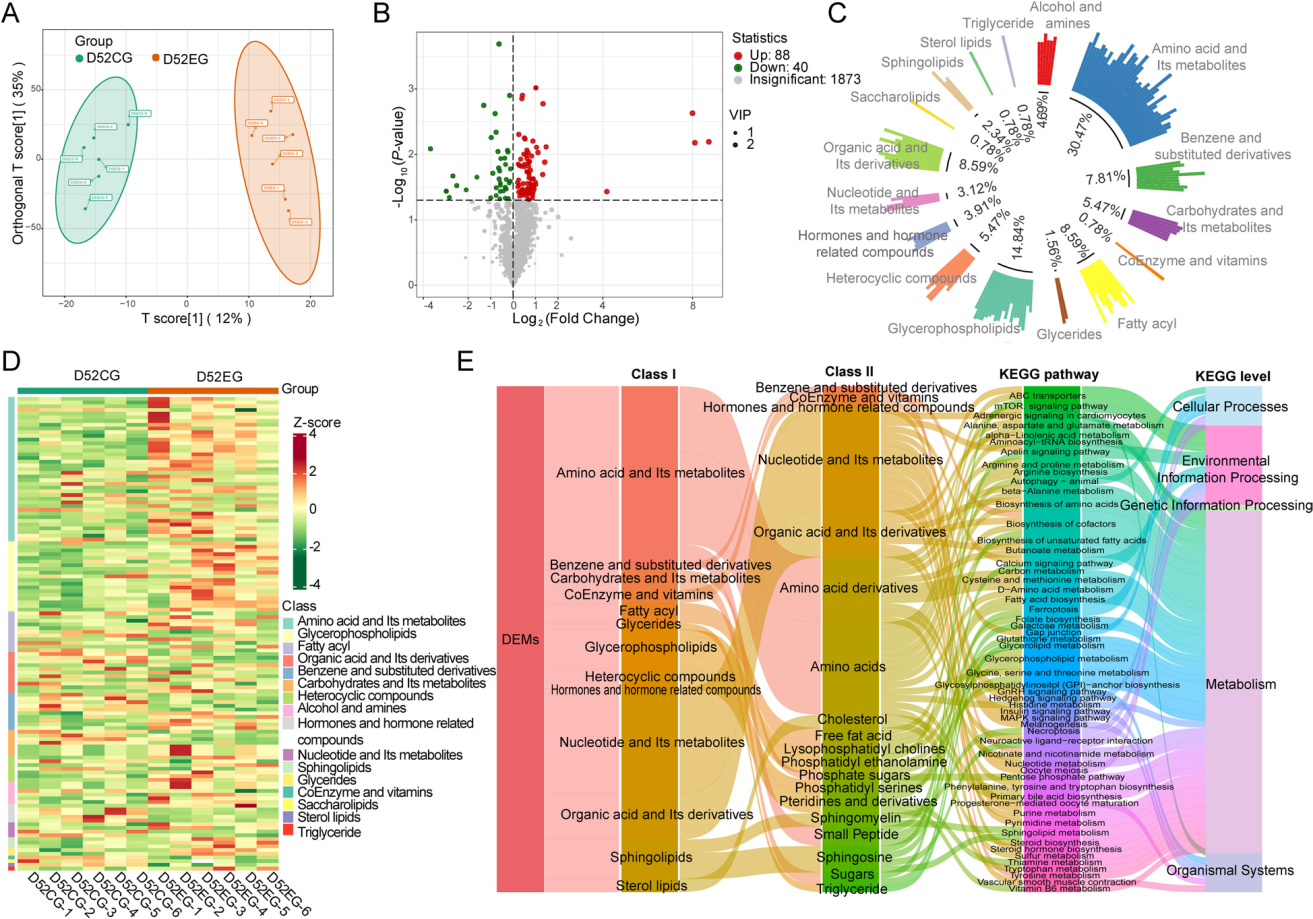
**Analysis of plasma DEM in day 52 EG (n = 6) and CG (n = 6) hens. A** OPLS-DA analysis of DEM shows a clear distinction between EG and CG hens. **B** Volcano plot of metabolites. Significant DEM (VIP > 1 and *P* < 0.05) are in red (upregulated) and green (downregulated), while others are colored in gray. **C** Circle chart of the proportion of primary classification of DEMs. **D** Heat map of DEM. Different colors are filled with different values obtained after standardization of relative content (red represents high content, green represents low content). **E** Sankey diagram shows classifications of differential metabolism, as well as KEGG annotations and KEGG classifications.

The OPLS-DA score plot also shows clear separation between EG and CG plasmas at 52 dpi (Figure 5A). Eighty-eight DEM were upregulated and 40 DEM were downregulated at this timepoint (Figure 5B). As at 10 dpi, the main categories with DEM were glycerophospholipids and amino acids and metabolites which accounted for 14.84% and 30.47%, respectively, of the altered substances (Figure 5C). The majority of DEM in EG plasma were increased (Figure 5D). KEGG annotation by pathway type suggests that the DEM at 52 dpi were associated mainly with metabolism, environmental information processing, organismal systems, cellular processes, and genetic information processing. The metabolic pathways that were involved centered on lipid metabolism, amino acid metabolism, carbohydrate metabolism, and metabolism of cofactors and vitamins. At the level of cellular processes, DEM mainly originated from autophagy, ferroptosis, necroptosis, gap junctions, and oocyte meiosis, whereas the calcium signaling pathway, apelin signaling pathway, neuroactive ligand-receptor interaction, ABC transporters, and mTOR, MAPK and Hedgehog signaling pathways were involved at the level of environmental information processing. The GnRH signaling pathway, progesterone-mediated oocyte maturation, and vascular smooth muscle contraction were changed in organismal systems, and aminoacyl-tRNA biosynthesis was affected in genetic information processing (Figure 5E). In summary, numerous plasma DEM were identified that are implicated in a range of cellular pathways and functions both 10 and 52 days after *M. synoviae* infection.

### Metabolites and pathways with the most pronounced differences after *M. synoviae* infection for ten and 52 days

**Figure 6:**
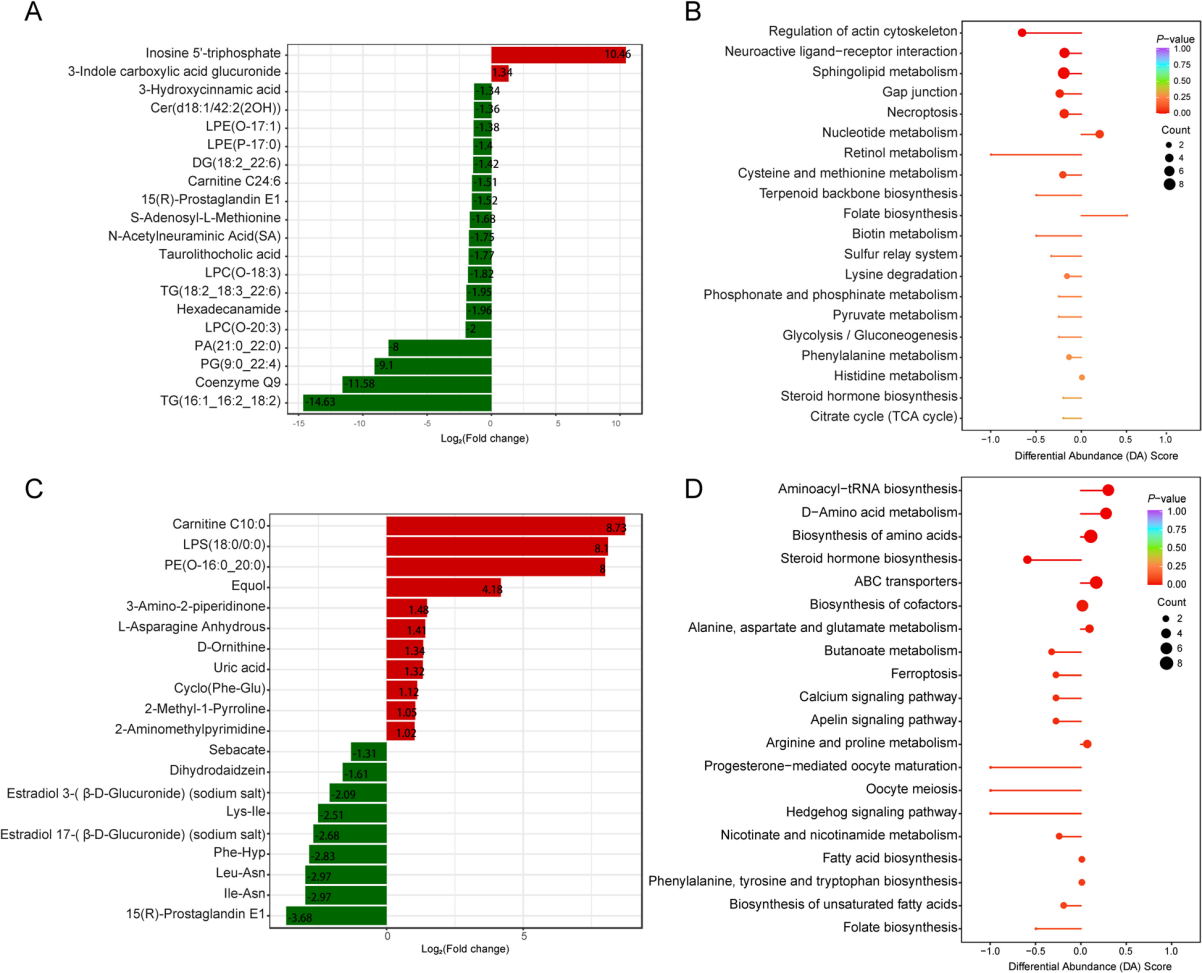
**Analysis of metabolomic signatures from hens after** ***M. synoviae* infection at days 10 and 52 showing the 20 most significant DEM and pathway enrichment.** **A** Twenty most significant DEM at day 10 after *M. synoviae* infection. The horizontal axis represents the log_2_FC of DEM, red represents an increase in metabolite content, and green represents a decrease in metabolite content. **B** Pathway enrichment of DEM at day 10 after *M. synoviae* infection. The vertical axis represents the differential pathway names (sorted by *P*-value) and the horizontal axis represents the differential abundance score. The differential abundance score reflects the overall changes of all metabolites in the metabolic pathway with a score of 1 indicating upregulation of the expression trend of all identified metabolites in the pathway, and a score of -1 indicating downregulation of the expression trend of all identified metabolites in the pathway. **C** Twenty most significant DEM at day 52 after *M. synoviae* infection. The horizontal axis represents the log_2_FC of DEM, red represents an increase in metabolite content, and green represents a decrease in metabolite content. **D** Pathway enrichment of DEM at day 52 after *M. synoviae* infection. The vertical axis represents the differential pathway names (sorted by *P*-value) and the horizontal axis represents the differential abundance score. The differential abundance score reflects the overall changes of all metabolites in the metabolic pathway with a score of 1 indicating upregulation of the expression trend of all identified metabolites in the pathway, and a score of -1 indicating downregulation of the expression trend of all identified metabolites in the pathway.

The most significant DEM 10 days after *M. synoviae* infection included 2 metabolites that were up-regulated (inosine 5'-triphosphate and 3-indole carboxylic acid glucuronide) and 18 down-regulated molecules (3-hydroxycinnamic acid, ceramide: cer (d18:1/42:2(2OH)), lysophosphatidylethanolamine (LPE): LPE (O-17:1), LPE (P-17:0), diacylglycerol (DG): DG (18:2_22:6), carnitine C24:6, 15(R)-prostaglandin E1, S-adenosyl-L-methionine, N-acetylneuraminic acid, taurolithocholic acid, lysophosphatidylcholine (LPC): LPC (O-18:3), LPC (O-20:3), triacylglycerol (TG): TG (18:2_18:3_22:6), TG (16:1_16:2_18:2), hexadecanamide, phosphatidic acid (PA): PA (21:0_22:0), phosphatidylglycerol (PG): PG (9:0_22:4), and coenzyme Q9). Subsequently, KEGG enrichment analysis shows that DEM at day 10 mainly were involved in regulation of the actin cytoskeleton, neuroactive ligand-receptor interaction, sphingolipid metabolism, gap junctions, and necroptosis (*P* < 0.05) (Figure 6B).

The most significant DEM 52 days after *M. synoviae* infection included 11 up-regulated metabolites (carnitine C10:0, lysophosphatidyl serines (LPS): LPS (18:0/0:0), phosphatidyl ethanolamine (PE): PE (O-16:0_20:0), equol, 3-amino-2-piperidinone, L-asparagine anhydrous, D-ornithine, uric acid, cyclo (Phe-Glu), 2-methyl-1-pyrroline, and 2-aminomethylpyrimidine) and nine down-regulated compounds (sebacate, dihydrodaidzein, estradiol 3-(β-D-glucuronide) (sodium salt), Lys-Ile, estradiol 17-(β-D-glucuronide) (sodium salt), Phe-Hyp, Leu-Asn, Ile-Asn, and 15(R)-prostaglandin E1) (Figure 6C). KEGG enrichment analysis shows that DEM mainly were enriched in aminoacyl-tRNA biosynthesis, D-amino acid metabolism, biosynthesis of amino acids, steroid hormone biosynthesis, ABC transporters, biosynthesis of cofactors, metabolism of alanine, aspartate, and glutamate, butanoate metabolism, ferroptosis, calcium signaling pathway, apelin signaling pathway, arginine and proline metabolism, progesterone-mediated oocyte maturation, oocyte meiosis, and the Hedgehog signaling pathway (*P* < 0.05) (Figure 6D).

### Common metabolomic signatures after *M. synoviae* infection

**Figure 7:**
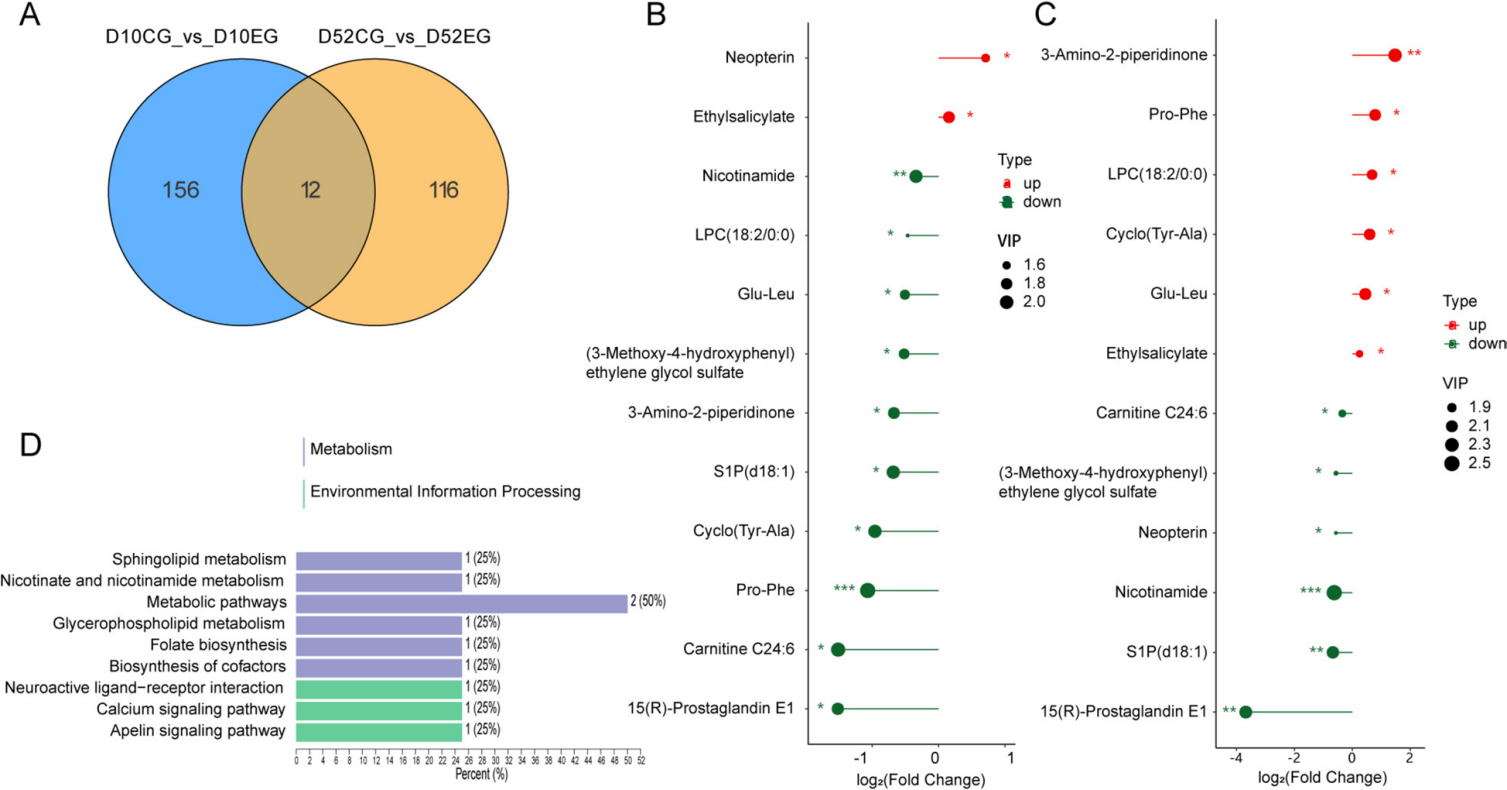
**Analysis of common metabolomic signatures from laying hens after* M. synoviae* infection at days 10 and 52.** **A** Venn diagram of co-expressed DEM between the groups. **B** Lollipop chart of day 10 changes in EG hens compared with CG hens. **C** Lollipop chart of day 52 changes in EG hens compared with CG hens. **D** KEGG pathway enrichment analysis for common DEM. The annotation results are classified according to the type of KEGG pathway.

Analysis of the overlap between differential plasma metabolites for day 10 EG compared to day 10 CG and for day 52 EG compared to day 52 CG revealed that 12 DEM were prominent in the two infection periods (Figure 7A). KEGG pathway analysis suggests that these DEM were enriched in folate biosynthesis, nicotinate and nicotinamide metabolism, sphingolipid metabolism, glycerophospholipid metabolism, biosynthesis of cofactors, apelin signaling pathway, calcium signaling pathway, and neuroactive ligand-receptor interaction (Figure 7D). Furthermore, among these DEM, six substances displayed the same expression trends at 10 and 52 dpi with a single upregulated metabolite (ethylsalicylate) and five downregulated compounds (nicotinamide, (3-methoxy-4-hydroxyphenyl) ethylene glycol sulfate, sphingosine-1-phosphate (S1P) (d18:1), carnitine C24:6 and 15 (R)-prostaglandin E1) (Figures 7B and C). ROC curve analysis shows that these six DEM had good performance in distinguishing *M. synoviae* infection. The AUC of these metabolites were > 0.8, with three DEM (nicotinamide, S1P (d18:1) and 15 (R)-prostaglandin E1) having AUC > 0.9 at both days 10 and 52 (Table 2). The six most prominent DEM may play significant roles in the pathogenesis of *M. synoviae* and have potential to become new therapeutic targets.

**Table 2:** **ROC analysis and logistic regression**

DEM	D10		D52	
	AUC	95% CI	AUC	95% CI
Nicotinamide	0.944	0.816–1.000	1.000	1.000–1.000
Ethylsalicylate	0.861	0.641–1.000	0.806	0.515–1.000
(3-Methoxy-4-hydroxyphenyl) ethylene glycol sulfate	0.861	0.625–1.000	0.833	0.554–1.000
Sphingosine-1-phosphate (S1P) (d18:1)	0.917	0.759–1.000	0.972	0.816–1.000
15(R)-Prostaglandin E1	0.917	0.738–1.000	0.944	0.816–1.000
Carnitine C24:6	0.944	0.816–1.000	0.833	0.580–1.000

ROC: receiver operating characteristic to evaluate the diagnostic capability of the model, AUC: area under the ROC curve, 95% CI: 95% confidence interval.

## Discussion

*M. synoviae* infection in the poultry industry causes considerable economic loss. Infection of laying hens and breeders with this pathogen has received considerable attention recently due to the impact on egg production and the mode of vertical transmission. Quality inspection of eggs and eggshells is crucial in the poultry industry. Inferior eggshell quality may result in damage and microbial infection which frequently leads to poor egg quality. These issues ultimately cause production losses and an increase in the embryo mortality rate [27, 28].

In this study, we conducted experimental infection of laying hens using *M. synoviae* MS-XS (Chicken/Hubei/4/2021) which is a Chinese isolate from a diseased chicken. Secondary infections with *Escherichia coli* (APEC, serotypes: O1, O2, O78) or IBV were excluded through PCR identification and antibody testing (Additional file 3). Egg production in infected hens was reduced 10 days after infection (*P* < 0.05) following which production dropped more rapidly (*P* < 0.01) (Figure 2D). Eggs with EAA first appeared at 14 dpi which was earlier than observed in experimental infections of Dutch [6] and Polish [12] chickens with *M. synoviae* when initial EAA manifestation began on 26 and 17 dpi, respectively, but was later than seen in studies of Italian hens [11] when EAA was evident 6 days after infection. Egg quality analysis here indicated an increase in eggshell weight and thickness whereas eggshell strength decreased markedly. The decrease in eggshell strength is consistent with the results of M. synoviae infection of Dutch chickens [6]. However, eggshell thickness results differ in the two studies, possibly because these two studies tested different eggshell segments and used different methods, and experimental hen breeds which inhibits comparability. Studies have shown that there is a low correlation between eggshell strength and thickness [29], with eggshell strength decreasing as the egg laying cycle lengthens, but eggshell thickness is randomly distributed [30]. Eggshell thickness and strength principally are affected by the time at which egg components passage through the shell gland (uterus), the deposition of eggshell components, and ultrastructure [31]. The ultrastructure of eggshells here (Figure 3C) suggested that abnormality of the mammillary body and the relative thickness of the palisade layer and mammillary layer impact eggshell strength [32, 33].

qPCR was used to determine the *M. synoviae* infection load which was concentrated mainly in the trachea, magnum, and uterus (Figure 2C) and correlated positively with ISH that was used to detect bacterial colonization and distribution (Figure 2F). The uterus is the main site of eggshell mineralization and provides inorganic minerals and organic matrix precursors required for mineralization. These factors are deposited in an orderly sequence to form specific eggshell microstructures [34, 35]. *M. synoviae* colonizes mainly in the uterus epithelium with severe pathological damage to the tubular glands (Figures 2F and G) that interferes with the synthesis and secretion of inorganic minerals and the organic matrix in the uterus. Moreover, *M. synoviae* affects ciliary motility in the oviduct (Figure 2G) which may induce changes in the uterine fluid content thereby affecting the deposition of calcium carbonate crystals [6, 36]. Further studies will reveal the mechanisms by which *M. synoviae* perturbs normal eggshell calcification.

Pathogen infection may alter the expression of host metabolites. The biosynthetic and metabolic capacities of parasitic forms of Mycoplasma are compromised due to a reduced genome size which makes these bacteria dependent on the host for diverse nutrients, including carbon sources, amino acids, inorganic salts, cholesterol, and fatty acids [37, 38]. LC–MS/MS provides the most advanced technology for selecting pathogen-derived metabolites with high stability and repeatability [39]. In this study, LC–MS/MS metabolomics analysis was used to probe host metabolic changes caused by *M. synoviae*. Most DEM, especially lipids and amino acids, had a downward trend at day 10 of infection (Figure 4D). Down-regulated lipid metabolites mainly comprised glycerophospholipids, glycerides, and triglycerides (Figure 6A) which may indicate that the proliferation of *M. synoviae* leads to impaired production of host precursor substances or metabolites in lipid metabolism and synthesis or may reflect increased lipid peroxidation in the host after *M. synoviae* infection. In addition, downregulation of antioxidant and anti-inflammatory effects substances, including 3-hydroxycinnamic acid, prostaglandin E1, coenzyme Q9, and taurolithocholic acid, suggesting an enhanced oxidative stress and inflammatory responses during infection. Reduced S-adenosyl-L-methionine levels also indicate changes in the pathological state of the host [40, 41]. The accumulation of inosine 5'-triphosphate, a metabolic intermediate involved in nucleotide synthesis, suggests that *M. synoviae* may satisfy replication by reshaping the purine and nucleotide metabolism pathways of the host [42]. N-acetylneuraminic acid, which is a prominent sialic acid, is utilized as a source of carbon and nitrogen by numerous pathogenic and commensal bacteria and is exploited for evasion of the innate immune system and recognition of entry into the host. Thus, down-regulation of N-acetylneuraminic acid correlates with proliferation and invasion by *M. synoviae* [43]. The main effects of *M. synoviae* infection after 10 days involved alteration of cell metabolic processes to promote survival and proliferation whereas high expression of glycerophospholipids and amino acids at day 52 suggests membrane fusion damage and dysregulated immunity [17]. In addition to downregulation of dihydrodaidzein and prostaglandin E1 that possess antioxidant and anti-inflammatory properties, the production of estradiol metabolites, including estradiol 17-(β-D-glucuronide) and estradiol 3-(β-D-glucuronide), was also dampened. Thus, changes in plasma metabolites at day 52 generally are contrary to those at day 10 which reflects changes in host pathological and physiological responses as infection progresses.

KEGG pathway analysis revealed that host necroptosis and ferroptosis pathways were enriched during *M. synoviae* infection. Mycoplasma-induced necroptosis is caused by autocrine TNF-α during infection [44] which is consistent with the upregulation of plasma TNF-α concentrations that were observed here. Ferroptosis is regulated by multiple cellular metabolic pathways, including redox homeostasis, mitochondrial activity and metabolism of amino acids, lipids and sugars, in addition to diverse signaling pathways relevant to disease [45]. *M. synoviae* infection may cause cell death by inflammation, oxidative stress, and by inducing an abnormal metabolic environment in host cells.

A combined metabolic analysis of plasmas from 10 and 52 dpi was conducted to identify the main pathogenesis modes of *M. synoviae* in hens. Sphingolipid metabolism and glycerophospholipid metabolism are co-enriched which suggests membrane fusion damage and immune damage induced by *M. synoviae* infection [17] which may be important features of pathogenesis. The lipid bilayer of cell membranes is susceptible to biomembrane fusion, and this structure requires the transcription of specific genes, cytoskeletal changes, and alterations in the nucleolus. Membrane fusion may also cause perturbations in receptor-identifying sites in the cell membrane which affects signal delivery between cells and the production of cellular factors [46]. Therefore, abnormalities in the apelin and calcium signaling pathways and neuroactive ligand-receptor interaction also may be implicated in pathogenesis by *M. synoviae*.

Ethylsalicylate, nicotinamide, (3-methoxy-4-hydroxyphenyl) ethylene glycol sulfate, S1P (d18:1), carnitine C24:6, and 15 (R)-prostaglandin E1 have distinct profiles in hens with and without *M. synoviae* infection which suggests that these factors may exert important roles in pathogenesis by this pathogen. The plasma metabolite ethyl salicylate was correlated positively with pro-inflammatory factors, including IL-6 and TNF-α, in elderly patients with chronic heart failure [47]. Consistently, we found here that this molecule was upregulated in plasma metabolism and also detected upregulation of IL-6 and TNF-α. The compound (3-methoxy-4-hydroxyphenyl) ethylene glycol sulfate is a major metabolite of the neurotransmitter norepinephrine. Downregulation of plasma (3-methoxy-4-hydroxyphenyl) ethylene glycol sulfate indicates disruption of norepinephrine metabolism. Norepinephrine has been reported to influence kisspeptin neurons and kisspeptin signaling to modulate gonadotropin-releasing hormone function in female rodents and in the human hypothalamus [48]. Carnitine assists in maintenance of cellular energy [49] and reduces oxidative stress [50] for proper oocyte growth and maturation of blastocysts, but also affects the hypothalamic-pituitary–gonadal axis to promote reproductive hormone secretory activity [51]. Thus, carnitine is potent in improving and/or restoring female reproductive functions [52]. Nicotinamide is a precursor in formation of nicotinamide adenine dinucleotide (NAD +). Both nicotinamide and its metabolic products are critical in essential cellular functions, including energy production and maintenance of genomic stability [53], stimulation of mitochondrial biogenesis which contributes to improved mitochondrial maintenance and function [54], and also is involved in a multitude of intra- and inter-cellular processes which regulate certain cellular metabolic, stress, and immune responses to physiological or pathological signals [55]. Sphingosine-1-phosphate acts as an intracellular and extracellular messenger that has important roles in modulating numerous cellular events including proliferation, differentiation, apoptosis, and inflammation [56, 57]. The molecule also has a role in maintaining cellular functions by regulating a series of complex signaling pathways. Prostaglandin E1 has diverse biological activities, including vasodilation [58], inhibition of intracellular reactive oxygen species, improvement of mitochondrial membrane potential, and inhibition of cell apoptosis [59]. In short, these six DEM are associated with energy and lipid metabolism, hormone abnormalities, and cell damage caused by *M. synoviae* infection which helps elucidate the mechanisms of *M. synoviae* pathogenesis and suggesting new treatment strategies that target these metabolites and the pathways in which these compounds intervene. However, its value in practical applications still needs further evaluation.

Alterations to KEGG enrichment in this study and other transcriptomics-related egg production analyses revealed that neuroactive ligand-receptor interactions and the calcium signaling pathway impinge on chicken egg production performance [60, 61]. The apelin signaling pathway also is involved in egg production [62]. Here, at day 52 of infection with *M. synoviae* which is the abnormal egg production stage, hormone and reproductive related pathways were also enriched, including steroid hormone biosynthesis, progesterone-mediated oocyte maturation, oocyte meiosis, and the Hedgehog signaling pathway, which suggests that these networks may also impact egg production. In addition, we believe that DEM melatonin, which was enriched at day 10, and cAMP, which was down-regulated at day 52, may play prominent roles in the decreased egg production rate and quality caused by *M. synoviae* infection (Additional file 4). It is well-documented that reproduction is controlled by complex endocrine interactions that involve the hypothalamus-pituitary–gonadal axis [63]. Melatonin has an inhibitory reproductive effect in long day animals [64, 65]. In addition, melatonin affects the production of hypothalamic monoamine and gonadotropin-releasing hormone by regulating the release of gonadotropins [66], and may affect cAMP and Ca2 + dependent intracellular mechanisms in the pituitary gland [67, 68]. However, the molecular pathways by which *M. synoviae* infection causes abnormal egg production in laying hens are mostly unknown. Further studies will aim to define better the direct effect of *M. synoviae* infection on abnormal egg production in laying hens, thereby helping to uncover the infection mechanism of *M. synoviae* and pinpoint new treatment strategies.

In conclusion, we confirm that *M. synoviae* colonizes the oviduct of laying hens and causes pathological damage that leads to a decrease in egg production rate and quality. This study also characterized the global plasma metabolism response to early and late stages of infection with *M. synoviae* in laying hens. *M. synoviae* infection induces diverse metabolic disorders, disrupts signaling pathways, and causes cell damage in the host. The common DEM identified at different infection time periods may serve as starting points for an improved understanding of the pathogenesis of *M. synoviae* and promote the search for new therapeutic targets for *M. synoviae* infection. Nevertheless, the changes observed in the experimental inoculation require additional validation in the natural progression of infection.

## Supplementary Information

**Additional file 1. Primers of APEC (O1, O2, and O78) and IBV**.

**Additional file 2. Display diagram of gross lesions in infected hens**. (A) Air sac lesions. CG hens had no air sac lesion whereas EG hens had severe air sac damage presenting as obvious thickening and with large accumulations of confined cheesy exudate. (B) Ovarian and oviduct lesions. Abnormal follicular development and oviduct atrophy were seen in EG hens. (C) Footpad swelling. Footpads of EG hens were swollen and contained pus when cut open. (D) Eggs with EAA. Eggs were produced with rough eggshells at the top beginning from day 14 of *M. synoviae *infection. (E) Deformed eggs from infected hens compared with control eggs.

**Additional file 3. Exclusion of secondary infections caused by** ***E. coli* and IBV**. (A) PCR detection of APEC (O1: 263 bp, O2: 355 bp, O78: 623 bp) in trachea and liver. M: 2000 bp DNA marker; N: negative control. (B) PCR detection of IBV (1600 bp) in trachea and uterus. M: 2000 bp DNA marker; N: negative control; P: positive control. (C) Detection of IBV antibody in serum.

**Additional file 4. Information on all metabolites corresponding to the grouping at days 10 and 52 after** ***M. synoviae* infection.**

**Additional file 5. Statistics of KEGG differential enrichment classification for days 10 and 52 after** ***M. synoviae* infection.**

## Data Availability

Datasets used and/or analyzed during the current study are available from the corresponding author on reasonable request.
