# Supplementary material for: Plasma metabolomics of Mycoplasma synoviae infection in SPF White Leghorn hens by liquid chromatography-tandem mass spectrometry
Source: Vet Res. 2025 Mar 22;56:65. doi: 10.1186/s13567-025-01494-z (PMC11929215; doi:10.1186/s13567-025-01494-z)
Supplement: Supplementary file 1 — Additional file 1. Primers of APEC (O1, O2, and O78) and IBV. [file 13567_2025_1494_MOESM1_ESM.docx]

**Additional file 1 Primers of APEC (O1, O2, and O78) and IBV.**

| Primer | Sequence (5’-3’) | Size of product (bp) |
| --- | --- | --- |
| ECO-F | CGATGTTGAGCGCAAGGTTG |  |
| ECO1-R | CATTAGGTGTCTCTGGCACG | 263 |
| ECO2-R | GATAAGGAATGCACATCGCC | 355 |
| ECO78-R | TAGGTATTCCTGTTGCGGAG | 623 |
| IBV-F | AAGCAGAGCCTTGTCCCG | 1600 |
| IBV-R | CATTTCCCTGGCGATAGAG |  |
