# Supplementary figures and images for: Plasma metabolomics of Mycoplasma synoviae infection in SPF White Leghorn hens by liquid chromatography-tandem mass spectrometry
Source: Vet Res. 2025 Mar 22;56:65. doi: 10.1186/s13567-025-01494-z (PMC11929215; doi:10.1186/s13567-025-01494-z)

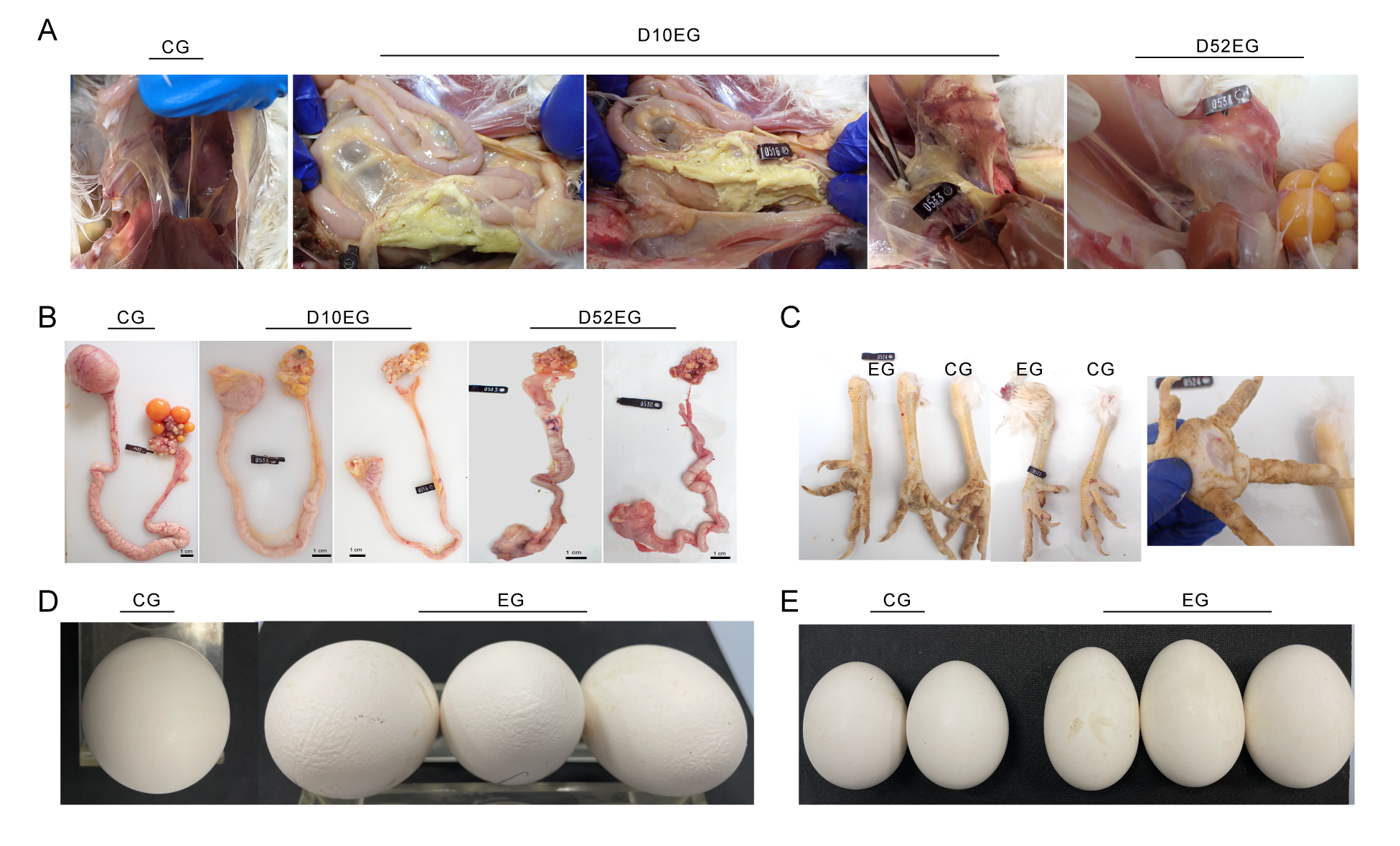

Supplement: Supplementary file 2 — Additional file 2. Display diagram of gross lesions in infected hens. (A) Air sac lesions. CG hens had no air sac lesion whereas EG hens had severe air sac damage presenting as obvious thickening and with large accumulations of confined cheesy exudate. (B) Ovarian and oviduct lesions. Abnormal follicular development and oviduct atrophy were seen in EG hens. (C) Footpad swelling. Footpads of EG hens were swollen and contained pus when cut open. (D) Eggs with EAA. Eggs were produced with rough eggshells at the top beginning from day 14 of M. synoviae infection. (E) Deformed eggs from infected hens compared with control eggs. [file 13567_2025_1494_MOESM2_ESM.tif]

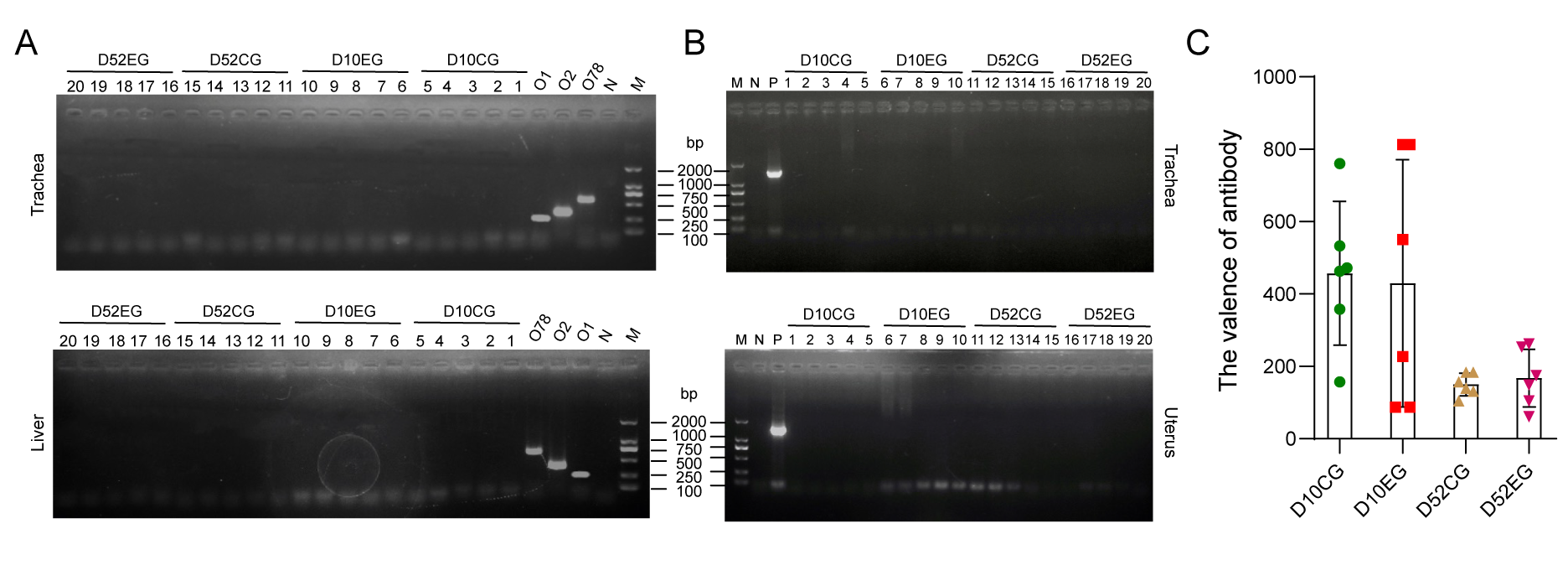

Supplement: Supplementary file 3 — Additional file 3. Exclusion of secondary infections caused by E. coli and IBV. (A) PCR detection of APEC (O1: 263 bp, O2: 355 bp, O78: 623 bp) in trachea and liver. M: 2000 bp DNA marker; N: negative control. (B) PCR detection of IBV (1600 bp) in trachea and uterus. M: 2000 bp DNA marker; N: negative control; P: positive control. (C) Detection of IBV antibody in serum. [file 13567_2025_1494_MOESM3_ESM.tif]
